# Perceptions of Change in Depression Among Participants in a Program Based on Permission Theory

**DOI:** 10.3390/healthcare14142191

**Published:** 2026-07-20

**Authors:** Fernanda Dias Alves, Jacqueline de Torres Boesso, Renato Pereira de Torres, Elton Euler da Silva Reis

**Affiliations:** Instituto de Pesquisa Permitir, São José dos Campos 12246190, Brazil; jacque.boesso@aliancadivergente.com.br (J.d.T.B.); renato.torres@aliancadivergente.com.br (R.P.d.T.); elton.euler@aliancadivergente.com.br (E.E.d.S.R.)

**Keywords:** depression, mental health, interpersonal relationships, complementary therapies, content analysis

## Abstract

**Background**: Depression is a major public health concern and remains a challenge despite traditional care approaches. This study sought to answer the following research question: how do participants in a program based on Permission Theory describe their perceptions of change related to the depressive experience? **Methods**: This exploratory and descriptive study employed a quantitative and qualitative approach, grounded in Bardin’s Content Analysis to analyze 23 spontaneous accounts from participants who reported experiences related to depression. Data were collected through self-reports voluntarily submitted by program participants through a routine administrative electronic form, characterizing a convenience sample. After thematic screening and manual verification of eligibility criteria, 23 accounts mentioning experiences related to depression were included in the analysis corpus. The participants evaluated their lives before and during the program. **Results**: The quantitative analysis showed an increase in self-reported scores of overall life evaluation during the program. Consistently, the qualitative accounts indicated that participants subjectively perceived changes in emotional, relational, and functional aspects of their everyday lives, including perceived changes in their depressive experience. **Conclusions**: These findings emphasize how participants interpret and describe changes in their emotional, relational, and functional lives, aspects that are often less visible in conventional mental health outcome research. These perceptions do not allow for inference of clinical effects or a causal relationship with program participation, reinforcing the need for controlled studies to investigate potential impacts on mental health outcomes.

## 1. Introduction

Depression is a global public health problem with a substantial impact on disability, morbidity, premature mortality, and suicidal ideation [1,2]. International estimates indicate that approximately 330 million people worldwide are living with depressive disorders [1]. In Brazil, data from the National Health Survey recorded approximately 17 million adults with a self-reported diagnosis of depression in 2019, with more recent estimates suggesting an increase to approximately 20 million in 2023 [3,4]. In addition to its clinical and social burdens, depression imposes substantial economic costs, exceeding one trillion dollars per year globally due to reduced productivity [5].

The high prevalence of depression and its social and economic impact intensify the challenge of providing effective and sustainable mental health care. Although pharmacological and psychotherapeutic treatments constitute the traditional foundation of care, a substantial proportion of patients continue to experience persistent symptoms, show a partial response, or undergo recurrences, making long-term remission difficult [6,7].

Nonpharmacological interventions—such as structured psychotherapies, physical activity programs, mindfulness-based practices, and psychosocial interventions—have demonstrated effectiveness across contexts; however, their effects tend to be heterogeneous in clinical practice due to factors such as adherence, access, intensity of follow-up, and suitability to individuals’ life contexts [8,9,10].

Among these approaches, Cognitive-Behavioral Therapy (CBT) stands out for its well-established empirical support [11,12]. A systematic review of randomized controlled trials with adolescents found that, among different CBT modalities, guided self-help showed the best results for depressive symptoms [11]. Systemic family therapy has also demonstrated efficacy for depressive symptoms in adolescents [13]. Psychoeducation, while not constituting a formal psychotherapeutic intervention, has also demonstrated efficacy in reducing emotional distress, including in non-clinical settings and with populations without a formal diagnosis [12].

Taken together, these findings suggest the limitations of exclusively intrapsychic approaches and reinforce the scientific relevance of complementary approaches targeting the relational dimensions associated with depression. In this context, innovative interventions aimed at changing how an individual relates to oneself and others and providing practical tools for reorganizing everyday life are particularly relevant. These dimensions have been highlighted by [14] as important in recovering from depression. Given this context, the present study sought to answer the following research question: how do participants in a program based on Permission Theory describe their perceptions of change related to the depressive experience?

Permission Theory is a conceptual model inductively developed by one of the authors from the systematic observation of behavioral patterns in personal development contexts (Section 2.1), still at an early stage of scientific validation, with no independent clinical or psychometric validation studies published to date. Permission Theory (PT) shares points of connection with approaches that consider individuals’ relational bonds, such as Family Systemic Therapy [15] and Cognitive-Behavioral Therapy [16]. However, it advances by proposing the existence of an unconscious utility of impactful events (such as accidents, betrayals, illness, or debt) in the individual’s life within a relationship, understood as phenomena that acquire a function in the relational context. In this model, the existence of the event is understood as conditioned by its relational utility, defined as the function the event serves in the dynamics between individuals, such that in the absence of this utility, the event does not configure as such.

This formulation of utility interfaces with the concept of secondary gain, traditionally described in the psychoanalytic literature as the indirect benefits associated with the maintenance of symptoms [17], and subsequently extended to include multiple meanings and functions in the clinical context [18,19]. However, PT differs by virtue of its specificity: the impactful event is understood in terms of its function in relational dynamics, being associated with obtaining a specific outcome in the relationship with the other. In secondary gain, the emphasis falls on indirect benefits or advantages derived from the maintenance of symptoms or conditions.

Although it does not constitute psychotherapy nor claim equivalence with models of proven efficacy, its assumptions find indirect support in established literature: the role of relational bonds in change is supported by studies on family functioning and depression [13,20]; the pattern identification and action-oriented decision-making structure is analogous to CBT [11]. Despite its exploratory nature, the PT formulation allows for the investigation of dimensions still underexplored in the literature, especially the articulation between relational function and the perception of change. The convergence between these assumptions and established literature justifies the use of PT as an interpretive framework in this exploratory study, provided its conceptual limits and the absence of independent clinical validation are respected.

Accordingly, the present study aimed to describe how, from participants’ perspectives, participation in a program based on Permission Theory was associated with perceived changes regarding depression through an analysis of spontaneous accounts.

## 2. Method

This study is an exploratory and descriptive investigation based on a qualitative approach grounded in Content Analysis (proposed by [21]) and complementary quantitative analyses. The quantitative analyses were used solely to contextualize self-reported perceptions and had no inferential or clinical purpose.

### 2.1. Permission Theory: Theoretical Framework

Permission Theory is a conceptual model for analyzing patterns of human behavior in relational contexts and personal development settings. Its central construct is internal authorization, defined as the individual’s explicit or implicit perception that a given change, action, or positioning is possible or permitted for oneself, considering the relational context in which the individual is embedded.

The central hypothesis of the theory is that it may be difficult to advance in certain areas of life when a desired future position is perceived as potentially incompatible with the preservation of existing relational bonds, roles, or configurations, thereby reducing or constraining the perception of internal authorization for change.

For analytical purposes, Permission Theory organizes observation into three interrelated axes: (1) event, defined as the observable occurrence or situation reported by the individual; (2) behavior, corresponding to patterns of response or action associated with the event; and (3) relationship, encompassing the relational bonds and contexts that may influence the maintenance, modulation, or alteration of these behavioral patterns. This structure enables behavior to be examined by simultaneously considering the event, the individual’s response, and the relational context in which both occur.

The model is applied considering both past events and situations that are ongoing or anticipated. The analysis of past events is methodologically facilitated by the fact that they are already consolidated as facts; however, their present relevance derives from the interpretive use made of them. The impact attributed to an event is not limited to its factual occurrence but extends to the function it comes to serve in current decisions and positionings.

Within this framework, events are examined according to their function within relational dynamics rather than being classified as positive or negative. An event may contribute to stabilizing relationships under tension or to reconfiguring positions among the parties involved. The connection between event and relationship is therefore understood as a central element in identifying observable regularities.

Progression across levels of permission entails the disruption of previously established patterns. These patterns may manifest as recurring events, repeated behaviors, or the maintenance of particular relational dynamics. The expansion of permission depends on the identification of such regularities and their corresponding reorganization. Attempts to transition to a new level of permission without a parallel adjustment in relational configurations tend to generate tension. Relational reorganization is thus a necessary condition for the consolidation of new outcomes.

Permission Theory (PT) proposes a 2 × 2 matrix of the relational utility of the impactful event, organized around two analytical dimensions: (a) the perceived relational consequence of the problem, expressed through the functions of sparing or forcing, and (b) its effect on the interpersonal bond, expressed through the functions of uniting or distancing.

According to the theory, these functions may operate outside the individual’s conscious awareness. In this context, forcing refers to the production of suffering for oneself or for the other; sparing refers to the avoidance of costs, responsibilities, or consequences perceived as undesirable; uniting refers to the strengthening of relational proximity; and distancing refers to the creation of distance within the relationship.

The combination of these two dimensions yields four possibilities of perceived relational utility of the impactful event, presented in Table 1.

This matrix can be constructed from relational patterns associated with emotional dependence [22], through protocols applied within the Permission Theory-based program, which allow for the identification and classification of the relational utility of the event.

The measurement of the event’s impact on the relationship can be made through an analysis of relational utility, in which the intensity of the impact is understood as an expression of this utility. The magnitude of the impact is directly connected to the utility in the relationship at that moment and is defined, unconsciously, in a way that ensures its relevance to the relational dynamics, neither too large to be endured nor too small to be ignored.

Conceptually, Permission Theory interfaces with fields that examine human behavior, personal positioning, and the influence of relational contexts on patterns of action, without formally aligning itself with specific psychological schools of thought. In this sense, it may be understood as engaging in dialogue with approaches that analyze behavioral change processes and relational dynamics in non-clinical contexts, while not claiming formal affiliation with any particular theoretical tradition.

Permission Theory (PT) engages with approaches that attribute centrality to relational bonds in the organization of human behavior. In Family Systemic Therapy, for example, the understanding that interaction patterns and symptoms may serve specific functions in maintaining the structure and equilibrium of the family system [15] is highlighted, as well as the notion that communication and behaviors acquire meaning in the relational context in which they occur [23]. In a complementary manner, Cognitive-Behavioral Therapy recognizes that interpersonal experiences influence the formation of beliefs and schemas, which guide the interpretation of situations and the emission of behavioral responses [16]. In this scenario, PT aligns with these perspectives by considering the relevance of relational bonds in understanding behavior, while advancing by introducing the hypothesis that impactful events may nurture an unconscious utility in the context of relationships. In PT, this utility is specific and associated with a specific outcome in the relationship with the other. In secondary gain, the emphasis falls on indirect benefits arising from the maintenance of symptoms or conditions [17].

A defining feature of Permission Theory is the centrality of relational dependence as a regulatory element of human advancement. The model proposes that the degree to which certain relational bonds are preserved influences the internal authorization an individual establishes to be, have, do, or go/be in new positions. This authorization functions as the organizing mechanism of change, determining the perceived legitimacy of sustaining new roles, outcomes, or conditions. In parallel, the theory introduces the concept of the functional utility of the problem, according to which recurring patterns may maintain relational configurations perceived as relevant, regardless of their objective verification. As long as a problem retains functional utility in regulating relational bonds, constraints on internal authorization are likely to persist.

Permission Theory was inductively developed from the systematic observation of behavioral and relational patterns in applied personal development contexts. Given this empirical origin, its hypotheses require further investigation to assess consistency, applicability, and limitations, thereby contributing to its theoretical refinement and scientific validation.

Although it was not originally formulated as a clinical or psychopathological model, its constructs may be used as an interpretive framework in studies investigating subjective experiences of change, provided that its conceptual boundaries and lack of diagnostic or therapeutic intent are respected. Although it does not constitute psychotherapy nor claim equivalence with models of proven efficacy, the theory’s assumptions engage with three established traditions. Regarding CBT, the PDA cycle (Perceive, Decide, and Act) is structurally analogous to the process of identifying dysfunctional patterns and action-oriented decision-making, even though CBT operates through formal clinical protocols and aims to modify cognitive schemas [11]. Regarding Family Systemic Therapy, whose efficacy for adults with depressive disorders was demonstrated in a meta-analysis of 30 RCTs [24], the connection is more direct: both conceive the reorganization of relational bonds as a necessary condition for the consolidation of new outcomes. This assumption finds empirical support in studies on family functioning and depression [13,20], although the theory does not claim direct derivation from these traditions.

In the context of the present study, Permission Theory was used exclusively as a conceptual framework to understand the self-reported accounts obtained through convenience sampling of participants in a structured personal development program, without implying clinical intervention, diagnosis, or therapy.

Additionally, the identification of participants included in the analysis was not based on diagnostic criteria for depression defined by the DSM-5-TR, ICD-11, or standardized psychometric instruments. Participants did not undergo clinical evaluation, nor did they complete validated scales for measuring depressive symptoms. Report selection was based on the identification of spontaneous references to depression or depression-related experiences within the testimonies submitted by participants. Accordingly, the focus of the analysis was on subjective perceptions and self-referenced narratives related to the depressive experience, rather than on the presence, severity, or remission of clinically defined depressive disorders.

This methodological approach is consistent with the exploratory and phenomenological nature of the study, whose objective was not to measure the prevalence, severity, or remission of depressive disorders, but rather to understand how participants described and narrated experiences that they themselves associated with depression. Consequently, the results should be interpreted as subjective accounts of experiences related to depression, rather than as clinical indicators of depressive symptomatology, diagnostic change, or therapeutic efficacy. This limitation should be taken into account when interpreting and generalizing the findings.

### 2.2. Description of the Program Based on Permission Theory

The program analyzed was developed based on Permission Theory, which underpins the operational model adopted in this study. Created in 2022, the program aims to offer a systematized structure for guidance and follow-up, focusing on the organization of everyday aspects related to health, relationships, and finances. Its central concepts include permission and emotional dependence, as defined in its theoretical formulation. The program consists of a set of structured activities, including classes, educational materials, and tools for routine organization, personal planning, and decision-making. It is delivered within an environment referred to as the Divergent Alliance, in which participants are embedded throughout the period of participation. Participants are organized into groups of approximately 30 individuals and supported by mentors who provide daily guidance through audio messages and weekly meetings. Each group also includes a host responsible for supporting new members, encouraging participation, and identifying difficulties reported by participants.

Daily interactions within the groups include reporting challenges, sharing experiences, and using structured tools. Among these tools is Think With Me, a standardized procedure in which the participant describes a specific situation and receives contributions from other members to organize thoughts, identify alternatives, and develop an action plan, in alignment with the PDA process (Perceive, Decide, and Act). Think With Me is applied exclusively within the program’s internal methodology and does not constitute a psychotherapeutic intervention.

The program also offers four structured protocols—Emotional Dependence, Emotional Protection, Fear, and Guilt—composed of stages that include the recognition of relational patterns, the establishment of interpersonal boundaries, and decision-making. In general, these protocols include stages of identifying problem situations, mapping the relationships involved, analyzing perceived impacts, defining interpersonal boundaries, reflecting on responsibilities, and elaborating concrete actions. At the end of the process, the participant is guided through a Perception, Decision, and Action (PDA) cycle, designed to facilitate the practical application of the knowledge and reflections developed during the protocol. A summarized description of the protocols is presented in Table 2.

In addition, the program uses the Marca Passos application, which centralizes lessons, tools, and individual records, allowing participants to track activities and revisit content. Among these tools is the Perfect Plan, an annual planning instrument oriented toward personal goals. Its construction is supported by recorded lessons and supporting materials, and participants may seek support from hosts or other group members whenever they deem it necessary.

The Perfect Plan begins with the definition of a broad objective (a “big dream”), based on which participants identify the life areas they consider priorities for achieving this outcome. One of the central purposes of the tool is to establish a clear reference for the desired future, allowing perceptions, decisions, and actions (PDAs) to be guided by the objectives to be achieved, so that the desired outcome becomes the primary reference for guiding the participant’s choices and behaviors, rather than present problems.

These areas include health, relationships/family, and finances/career, and participants may concentrate their efforts on one, two, or all three dimensions. To assist in this prioritization, participants distribute five tokens among the different domains, allocating greater attention to the areas perceived as more fragile or limiting to the achievement of their objectives. Based on this prioritization, goals, concrete actions, progress indicators, and execution deadlines are established. The plan is reviewed quarterly in meetings conducted by mentors, aimed at reviewing the established goals, assessing progress achieved, and guiding the next steps.

**Figure 1:**
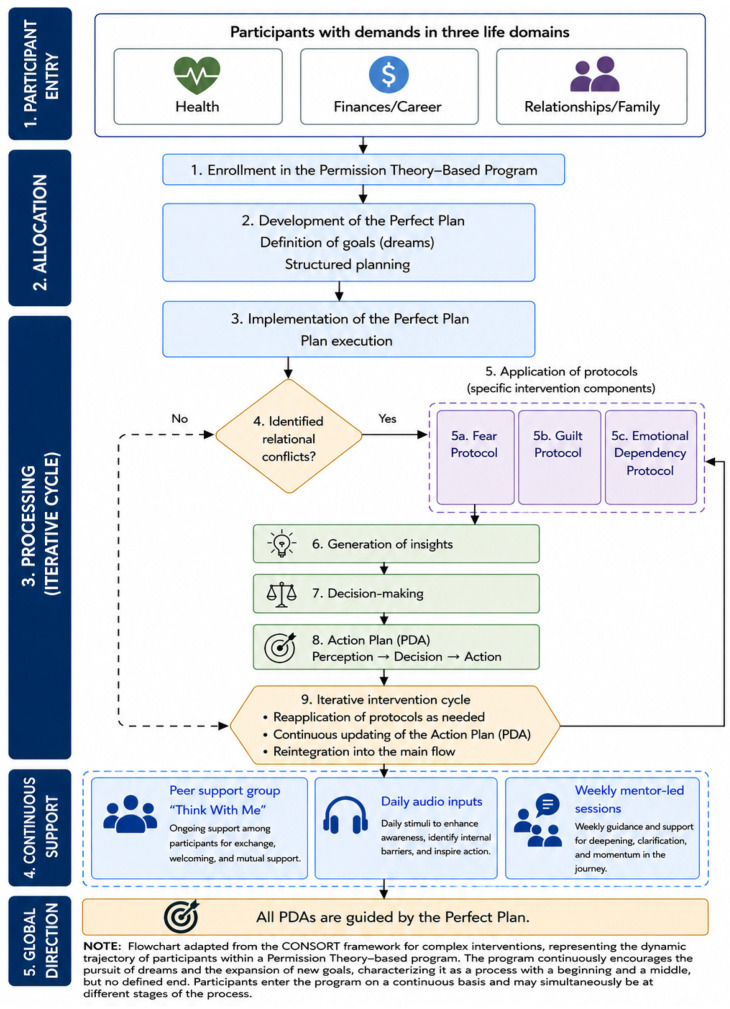
Flow of participation in the Program Based on Permission Theory (adapted from the CONSORT model for complex interventions). Source: elaborated by the authors.

The program’s environment is organized to promote support among participants, self-responsibility, and continuous monitoring of planned activities, providing a structured dynamic of monitoring and guidance. This description is provided solely to contextualize the environment in which participants’ reports were produced. Participant enrollment occurs in a continuous flow, with no formation of closed cohorts or recruitment waves for the implementation of the Program. The Program has no predetermined duration, being conceived as a continuous process of personal development in which participants define goals, implement action plans, apply specific protocols when necessary, and iteratively review their goals. Accordingly, the length of participation varies according to each participant’s needs and engagement. This model of continuous-flow participation and indeterminate duration reflects the conception of the Program as an iterative personal development process, guided by individual goals rather than by a protocol of sessions with a fixed number. The overall flow of participation in the program is illustrated in Figure 1.

From the perspective of its placement within the spectrum of non-pharmacological interventions, the program shares with psychoeducation the logic of structuring knowledge and tools to promote changes in perception and behavior without constituting formal psychotherapeutic intervention. A recent study demonstrated that structured psychoeducational interventions, even conducted in non-clinical settings, produced significant reductions in emotional distress [12].

## 3. Participants and Data Collection

The data analyzed in this study were derived from spontaneous accounts submitted by program participants through an electronic form routinely used for administrative purposes. The form remained continuously available to participants throughout the entire study period, allowing accounts to be submitted at any point during participation. There were no predefined collection points, specific Program milestones, or time-based criteria for completing the form. Between November 2024 and July 2025, 5094 responses were received, of which 395 had been previously archived by the program’s management team for routine administrative purposes before being made available to the researchers for analysis. It is acknowledged that this pre-selection was carried out by a team with an institutional affiliation to the evaluated program, which constitutes an additional limitation to the transparency of the corpus formation process and a potential conflict of interest that should be considered when interpreting the findings. No information was recorded regarding participants’ length of time in the Program at the time their accounts were submitted.

The form contained 14 open-ended questions covering basic sociodemographic information (such as age, gender, marital status, and occupational status) and questions related to the program, including descriptions of life prior to entry, perceived results, strategies used, and the impact of the activities. Responses to these questions were analyzed in this study.

To identify accounts that directly mentioned experiences associated with depression, initial thematic screening was conducted using the term “depress”. After filtering and manual verification of the content, 23 accounts met the inclusion criteria and constituted the corpus of qualitative analysis.

Duplicate accounts, very brief responses (such as emojis or one-word messages), automated responses, offensive content, or references exclusively to third parties were excluded. This secondary dataset was provided by participants within the routine context of the program without any researcher intervention.

Of the 5094 reports spontaneously submitted by Program participants during the analyzed period, 395 comprised the testimony bank made available for analysis. The administrative criteria for inclusion in this bank were: (a) presence of a narrative description with sufficient length and detail to characterize a personal experience, excluding responses composed exclusively of emojis, one-word messages, or non-narrative content; (b) explicit authorization from the participant for public use of the testimony; and (c) the participant’s agreement to record their account in interview format.

The application of these criteria was carried out routinely by the Program’s management team for administrative and institutional communication purposes, prior to the conception of the present study. As the team responsible for this process had an institutional affiliation with the evaluated program, it cannot be ruled out that the archiving of reports may have been influenced, even if unintentionally, by the nature or favorability of the testimonies. This potential selection bias should be considered when interpreting the findings.

Thus, the reduction between the total number of responses received and the 395 reports available for analysis reflected administrative and operational eligibility criteria of the program, not involving formal criteria related to the thematic content of the reports or the presence of specific outcomes. However, since the reports not included were not analyzed for the purposes of this study, it is not possible to completely exclude the possibility of selection bias at this stage. Even so, it is acknowledged that this stage may have introduced self-selection bias, since only participants willing to publicly share their experiences were included in the testimony bank made available for analysis.

**Figure 2:**
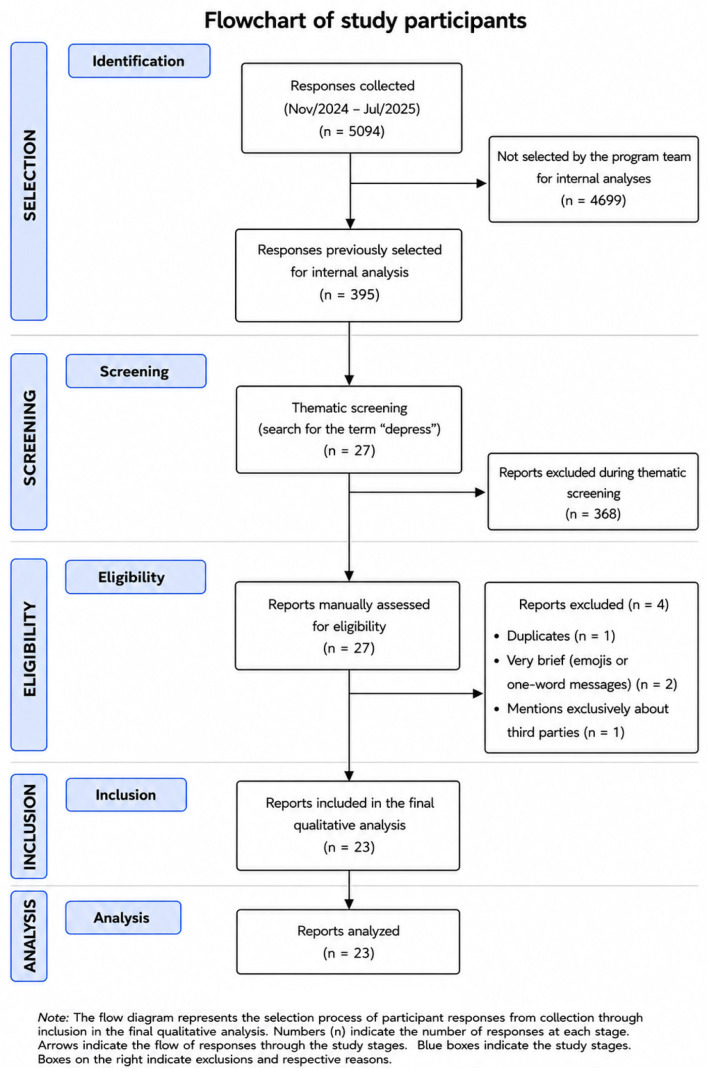
Flowchart of participants included in the study (adapted from the PRISMA model). Source: elaborated by the authors.

Figure 2 illustrates the complete flowchart of the process of identification, screening, eligibility assessment, and inclusion of participants in the study.

## 4. Data Analysis

### 4.1. Quantitative Analyses

Categorical variables were described based on their frequency distributions (absolute and relative), whereas numerical variables were represented by measures of central tendency (mean and median) and variability (standard deviation, minimum, and maximum values).

The “life evaluation score” variable was obtained through two self-reported questions retrospectively applied to participants: “How would you rate your life in general BEFORE Aliança Divergente?” and “How would you rate your life in general AFTER Aliança Divergente?”. Responses were recorded on a scale from 0 to 10, with higher values indicative of a more positive overall evaluation of one’s own life. This is a single-item administrative measure used for internal monitoring of the Program. Although the structure of the measure is similar to that used in the Cantril Self-Anchoring Striving Scale, it does not constitute a formal adaptation of that instrument, and it has not been submitted to a psychometric validation process. Its use in this study has a descriptive and exploratory purpose, without claiming equivalence to validated instruments of subjective well-being. The normality of the distribution was assessed using the Shapiro–Wilk test, which indicated an absence of normal distribution. Given this, non-parametric tests were used.

To compare the scores assigned before and during participation in the program, the Wilcoxon test was applied with a significance level of 0.05. Additionally, a box plot was constructed to visualize these distributions.

Differences in scores were also explored according to sociodemographic characteristics (sex, age group, marital status, and the pillar with the best results). The Mann–Whitney test was used for binary comparisons (sex), and the Kruskal–Wallis test was employed for variables with more than two categories. These analyses were included to examine possible variations in self-reported perceptions across subgroups. Statistical analyses were conducted using SPSS software, version 26.0. The results are exploratory in nature due to the small sample size.

### 4.2. Qualitative Analyses

The qualitative analysis was conducted according to Bardin’s Content Analysis [12]. Initially, a pre-analysis was carried out through a floating read of all of the accounts. Subsequently, material exploration was performed through independent reading and thematic coding. Two context units were identified: 1. before participation in the program and 2. during participation in the program. For each context unit, four registration units were identified. From these, categories emerged, which were grouped by semantic similarity, as presented in Table 3.

In the subsequent treatment and interpretation stage, representative statements from each category were highlighted. The initial coding was conducted by one of the researchers through inductive thematic analysis carried out in electronic spreadsheets. Subsequently, the proposed codes and categories were reviewed by two additional researchers, who examined the correspondence between the selected excerpts, the assigned codes, and the constructed thematic structure. This process aimed to critically evaluate the initial interpretations, verify the consistency of the relationship between the data and the thematic categories, and reduce the influence of exclusively individual perspectives in the organization of the results.

Participants were identified using alphanumeric codes (P1, P2, P3…) to preserve anonymity. The statements were maintained as reported, with correction only of evident typographical errors.

As a reflexivity statement, it is acknowledged that the researchers had proximity to the studied context and an institutional affiliation with the analyzed program, which may constitute a potential source of interpretive bias. The review carried out by the additional researchers included the analysis of the adequacy of the codes to the selected excerpts and the qualitative discussion of the proposed interpretations, seeking to reach consensus regarding the thematic organization of the data. It is acknowledged, however, that this procedure did not include blind and independent recoding of the data, calculation of an inter-rater agreement coefficient (such as Cohen’s kappa), or formal analytic audit—more rigorous methodological reliability procedures that were not adopted in this study and that constitute limitations to be considered when interpreting the results.

## 5. Use of Artificial Intelligence

Writing and revision were supported by a research assistant using artificial intelligence (GPT-5, OpenAI), which was employed to enhance the clarity and consistency of scientific writing. Qualitative analysis and data interpretation were conducted entirely by the researchers, in accordance with Bardin’s methodology [12]. The AI tool did not perform any analysis.

## 6. Ethical Aspects

It is relevant to highlight that the analyzed program does not propose to clinically treat depression, nor does it replace psychiatric or psychotherapeutic follow-up. All participants whose accounts mentioned medication use or professional follow-up maintained these forms of care in parallel with the program. The use of non-clinical structured interventions in contexts of emotional suffering raises ethical questions that deserve consideration. The literature recognizes the feasibility of non-clinical psychoeducational and behavioral interventions for emotional suffering [12], provided that their limits are clearly defined for participants. The program based on Permission Theory explicitly states, in its materials, that it does not constitute a psychotherapeutic intervention. However, the absence of prior clinical screening and the possibility that participants with severe clinical depression may turn to the program as a substitute, rather than a complement, to formal treatment represent an ethical risk that future studies should systematically monitor.

All data were handled anonymously and confidentially in accordance with the Brazilian General Data Protection Law (Lei Geral de Proteção de Dados—Law No. 13,709/2008) and the ethical principles of scientific research.

## 7. Results

The study included 23 individuals with ages ranging from 29 to 62 years (mean of 44.5 years; standard deviation of 9.5 years). Most participants were female (78.3%), married (52.2%), and residents of the southeast region (47.8%). The remaining sociodemographic characteristics are presented in Table 4.

Regarding the life classification variable, before participation in the program, the mean was 3.1, whereas it increased to 8.7 during the program. The description of the results is exclusively self-reported, and the mean difference observed between the two time points was 5.5 points and statistically significant (*p* value = 0.000) (Table 5). The median also showed a substantial increase, rising from 3 to 9, indicating a shift in the distribution toward higher levels.

**Figure 3:**
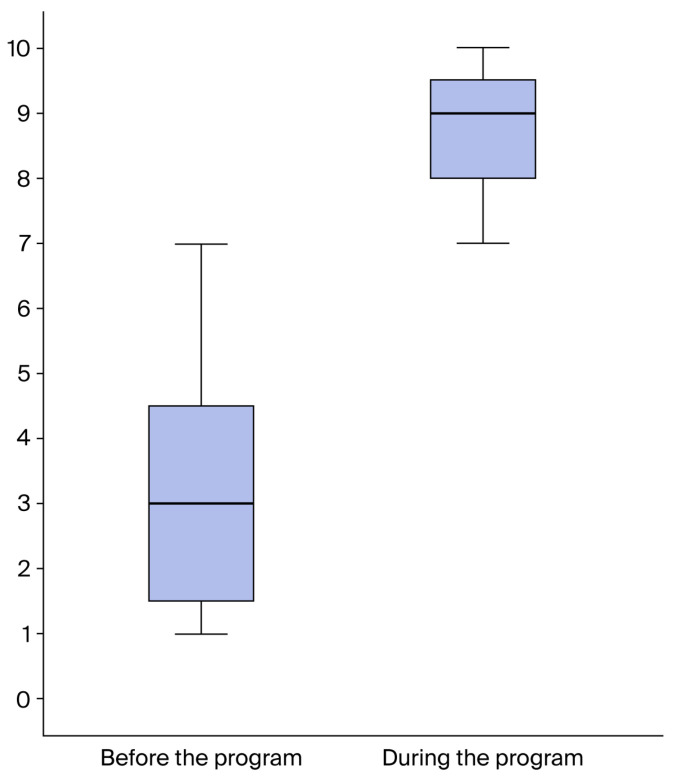
Box plot of life classification scores (before and during participation in the program). Source: elaborated by the authors.

Figure 3 presents the quartiles of life classification levels before and during participation in the program. The median increased from 3.0 to 9.0, reflecting a consistent increase in the self-reported evaluation of one’s own life.

Table 6 presents the results of the comparison of differences in life classification levels in relation to sociodemographic characteristics. No comparison showed a statistically significant difference, indicating that the observed increase was similar across the analyzed subgroups. These findings should be interpreted with caution given the small sample size and the low statistical power of stratified analyses. As noted in the literature, a larger sample could reveal how moderating variables, such as age and marital status, influence themes such as financial instability and emotional dependence. Future studies with greater statistical power and inclusion of standardized baseline screening instruments could allow more precise comparisons between subgroups.

Finally, no corrections for multiple comparisons were applied, given the exploratory nature of the analyses. The results represent self-reported perceptions rather than clinical diagnostic measures.

### 7.1. Qualitative Analysis of Participants’ Accounts Prior to Entry into the Program

Based on the analysis of participants’ self-reports before entry into the program, four registration units were identified, from which seven main categories emerged, reflecting the most recurrent and relevant aspects of the reported experiences (Table 1). These categories were organized according to the frequency of accounts, from the most to the least recurrent, allowing for a structured presentation of the main aspects mentioned by participants regarding the difficulties experienced prior to participation in the program.

### 7.2. Financial Instability and Precarious Work

This category emerged as the most prominent among the accounts, mentioned by 17 participants, evidencing perceptions of indebtedness, economic instability, and feelings of helplessness in relation to work, which was experienced more as overload and frustration than as a source of personal fulfillment.

The statements also reveal a context of economic instability perceived by participants, in which constant efforts to remain financially stable did not result in desired stability: “Working a lot, earning little, without acquiring assets…” (P01). In some cases, the reported financial situation included loss of assets and risk of housing insecurity: “Completely indebted… I lost my car, I was almost evicted…” (P14). Statements such as “Totally bankrupt, indebted…” (P18) reinforce the perception of significant financial difficulties, frequently accompanied by expressions of discouragement reported by the participants themselves.

### 7.3. Perceptions Related to Depression

This category emerged as the second most frequently mentioned, present in 15 accounts, in which participants reported situations that they related to depression, as well as references to anxiety, insomnia, discouragement, and thoughts associated with death.

Prior to participation in the program, participants reported experiences characterized by intense fatigue, feelings of discouragement, and difficulty performing everyday activities, as expressed in their narratives: “I wanted to die, my real desire was not to be alive anymore… I wouldn’t get out of bed. Total depression” (P17).

The persistence of depressive symptoms over time was also highlighted: “I had been in deep depression since the end of 2023…” (P23). In addition, use of antidepressant medication was observed: “I took antidepressant medication to be able to endure the weight of life” (P02).

### 7.4. Conflictual Interpersonal Relationships and Lack of Boundaries

This category was mentioned by 13 participants, emerging as a relevant aspect of experiences prior to entry into the program. The statements describe perceived difficulties in everyday interactions, often marked by conflict and poorly defined boundaries.

Participants reported situations in which they perceived themselves to be in conflict and had difficulty refusing the demands of others: “Living with many family conflicts for not knowing how to relate, for not knowing how to say no” (P06). Challenges in marital relationships and relationships with family of origin were also mentioned: “My marriage was terrible… we fought a lot… I prioritized my mother at his expense…” (P02). In addition, some participants used strong expressions to characterize these experiences: “I was in an abusive relationship, in a war… with my mother” (P13).

### 7.5. Absence of Purpose and Life Perspectives

This category was mentioned by 12 participants and describes a perceived lack of direction, plans, and goals for the future. The statements indicate that participants perceived their lives as lacking prospects and marked by burden and discouragement: “Without a life plan and purpose…” (P01).

This perception also appears in other accounts that refer to the absence of direction or clarity regarding next steps: “My life felt heavy and meaningless…” (P02); “Not knowing where life was going” (P05). These expressions reflect how participants described their relationship with the future, characterized by uncertainty and low expectations.

### 7.6. Impaired Physical Health and Risk Behaviors

This category was reported by ten participants, who described difficulties related to physical health care, including periods of sedentary behavior, dysregulated eating, weight gain, and recurrent episodes of illness.

Some participants mentioned perceptions of fragile health and persistent physical symptoms: “Without health…” (P18); “Becoming ill constantly…” (P01); “…my whole body inflamed…” (P21). Difficulties involving eating behaviors and regular physical activity were also reported: “Food compulsion every day” (P17); “Sedentary… weighing 130 kg…” (P22). These descriptions reflect how participants perceived their physical condition prior to entry into the program.

### 7.7. High Emotional Dependence and Lack of Permission

This category was identified in seven accounts in which participants explicitly mentioned the terms “emotional dependence” and “lack of permission”. In addition to these explicit mentions, elements related to these themes also appeared in other accounts, indicating that such experiences were perceived as present in different situations prior to entry into the program.

Participants described feelings of fear and perceptions of difficulty in living their lives with autonomy, associated with the expressions they themselves used: “…lack of permission and deeply ingrained fears” (P10); “In relationships, everything was filled with emotional dependence…” (P20); “…with many fears, a lot of emotional dependence” (P03). These statements reflect how participants described their experiences prior to participation in the program.

### 7.8. Low Self-Esteem and Negative Self-Evaluation

This category was reported by six participants, who described perceptions of dissatisfaction with themselves, accompanied by feelings of inadequacy and negative self-evaluation.

The accounts included expressions of self-devaluation and descriptions of discontent with one’s own image: “Feeling ugly, inferior…” (P01); “I was unhappy and felt like a failure every day” (P02). Perceptions of a lack of meaning in everyday life were also mentioned: “I thought, what is the point of all this…” (P09).

Prior to participation in the program, participants reported a set of challenges distributed across the three pillars—finances, relationships, and health—in which experiences related to depression appeared centrally. The description of these initial conditions made it possible to contextualize the accounts and to establish a point of comparison for the perceptions presented during participation in the program. The subsequent stage of the analysis focused on the accounts produced during the program, which made it possible to systematically examine the changes perceived by participants over the course of their trajectory.

### 7.9. Perceived Transformations During the Program

The qualitative analysis of the self-reports during program participation identified the same registration units found in the analysis of self-reports before entry into the program (Table 4). However, the emerging categories revealed self-perceived changes across the three pillars addressed in the program: health, family/relationships, and finances/career. Seven categories were identified, organized from the most to the least recurrent: (1) reconstruction of relational stance and self-care; (2) financial and professional improvement; (3) perceptions of reduction of the depressive experience; (4) increased permission and decreased emotional dependence; (5) sense of support and direction; (6) understanding of the usefulness of illness; and (7) planning and future projection.

These categories make it possible to understand, in an integrated manner, the aspects highlighted in the accounts as part of the changes perceived during the program, encompassing the three pillars addressed in the program: health, finances, and relationships.

### 7.10. Reconstruction of Relational Stance and Self-Care

This category was mentioned by all participants. The accounts describe perceived changes in the way interpersonal relationships are conducted and how participants organize their own routines. Participants reported having begun to establish boundaries, reorganize family ties, and adopt a stance perceived as more active in relation to everyday life: “Setting boundaries and having a new relationship with our families of origin” (P07); “…I now have strong relationships with clear boundaries among friends and family” (P16); “I am a new woman, with a different stance, I know myself more, I respect myself” (P02).

The statements also indicate greater self-accountability for one’s own actions and adherence to practices described as oriented toward self-care and discipline: “I stopped blaming people and the world for the results I didn’t have” (P01); “I joined the gym… I train five days a week” (P05). In addition, some participants mentioned the reorganization of time and daily activities: “I have time to spare, I am building strong relationships with clear boundaries” (P06).

### 7.11. Improvement in Financial Stability and Professional Performance

This category was mentioned by 17 participants, with accounts describing perceived changes in financial organization, economic stability, and work.

The statements included references to the reorganization of finances: “I organized my financial life…” (P06); the repayment of debts and acquisition of assets: “…I paid off all the debt I had, I bought a car…” (P13); and improvements in professional activity: “I gained good clients, I am prospering financially” (P12).

### 7.12. Perceptions of Reduction of the Depressive Experience

This category was explicitly identified in 13 accounts. The statements describe self-reported perceptions of a reduction of the depressive experience, accompanied by mentions of a greater sense of well-being, as reported by participants themselves during their participation in the program.

Participants reported changes they perceived as substantial, including references to the reduction or discontinuation of medication use: “I improved my mental and physical health, I stopped taking medication for depression and anxiety…” (P13); “…I was cured of depression, I was discharged from psychotherapy within a few months” (P11). In some cases, participants mentioned perceiving rapid improvement: “I came out of depression in the first month of AD (Program)” (P3). Sensations of relief and a resumption of vitality were also described: “I feel lighter, the symptoms of depression stopped…” (P15).

### 7.13. Increased Permission and Decreased Emotional Dependence

This category was explicitly mentioned by 11 participants, who used terms such as “permission” and “emotional dependence” to describe their experiences. Accounts in which these themes appeared indirectly were also identified.

The statements indicated perceptions of change related to the way participants experienced fear, dependence, and previously described limitations, as well as mentions of increased permission: “I increased my permission…” (P17); “…emotional independence and putting an end to the fears that paralyzed me” (P06); “…I lived in an emotional prison, today I live free…” (P09). Some participants also mentioned perceived effects on family relationships: “…I had a lot of emotional dependence, and by freeing myself from this, I also freed my daughter and my husband from the restraints this entailed…” (P21).

### 7.14. Feeling of Support and Direction

This category was mentioned by seven participants, who described a feeling of support and receiving guidance during participation in the program. The statements highlighted support from the group and interactions throughout the process: “Now I feel supported. Through the group allies, I feel more confident” (P06); “…I felt supported, no longer alone” (P17). Changes perceived after receiving guidance were also mentioned: “They changed my confidence, now with correct guidance…” (P08).

### 7.15. Understanding the Usefulness of the Illness

This category was explicitly mentioned by five participants, who used the terms “usefulness of the illness” or “usefulness of depression” to describe their experiences during the program.

In these accounts, participants stated that they had recognized, in their own perception, aspects that they associated with the way they experienced depression: “…I discovered the usefulness of depression…” (P09); “That was the usefulness of my illness…” (P23); “…I discovered the usefulness of the illness” (P02).

### 7.16. Planning and Future Projection

This category was mentioned by four participants, who reported the resumption of the ability to plan, dream, and establish personal and professional goals. The statements describe a movement toward practical reorganization of everyday life and an expansion of perspectives regarding the future, accompanied by perceptions of greater confidence and clarity about what they wished to achieve.

The accounts indicate the gradual reconstruction of personal and professional projects, expressed in the formulation of plans and the resumption of desires: “…I created a plan, I got organized, and it made me want to believe in my dreams again” (P12). In addition, some participants reported perceptions of expanded possibilities and strengthened confidence in their own trajectory: “Today I started living again, wanting again, dreaming again, planning again, having big dreams” (P23); “I have never been so confident about our progress” (P08).

In summary, the analyzed accounts revealed consistent differences between the period prior to and the period during participation in the program, as described by participants in aspects related to health, relationships, and finances.

## 8. Discussion

The results of this study showed that, in the participants’ perceptions, participation in the program based on Permission Theory was associated with a change in their depressive state. In the quantitative analysis, a consistent improvement was observed in participants’ evaluations of their lives overall during the program, a pattern that remained stable across all sociodemographic subgroups analyzed. Consistent with these findings, the qualitative analysis revealed that prior to participation, the accounts contained descriptions of marked depressive symptoms, accompanied by financial instability, relational difficulties, low self-esteem, patterns of emotional dependence, and lack of permission, as well as an absence of future perspectives. During participation, these same participants began to report a reduction in their experience of depression, improvements in interpersonal relationships and self-care, increased permission and reduced emotional dependence, financial and professional organization, and a resumption of planning and future projection. These elements were related to a greater capacity to shape aspects of their own lives that they had previously described as limited.

The articulation between the quantitative and qualitative findings demonstrates that for the participants, the program was associated with perceived changes in depression and suggests that these changes did not occur in isolation but rather constituted a perceived movement of broader reorganization of their lives. When comparing the two moments (before and during program participation) consistency was observed in the records, a fact that guided the use of the same names in the registration units. However, it is important to highlight the self-reported improvement across the three pillars addressed in the program: health, family/relationships, and finances/career, which reinforces the importance of the method for capturing nuances of transformation spontaneously reported by participants. This contrast between the “before” and “during” experiences highlights the relevance of examining subjective dimensions of change in studies focused on depressive symptoms.

These findings can be contextualized in light of the literature on non-pharmacological interventions and relational factors in depression. The changes perceived by participants in the relational, emotional, and functional domains are consistent with evidence demonstrating that family functioning and the quality of interpersonal bonds are significant predictors of emotional well-being in adults [13,20]. In particular, participants who reported relational changes, such as establishing boundaries and reorganizing family bonds, may have experienced subjective improvements consistent with evidence that family cohesion and adaptability are protective factors against depression [20,25]. From the perspective of Permission Theory, relational reorganization is understood as a necessary condition for the consolidation of new outcomes, an assumption that finds empirical support both in family systemic therapy [24] and in studies on family functioning [20].

Participants reported feeling supported and guided on an ongoing basis, suggesting that this supportive environment was relevant to the changes they perceived in relation to depression. This feeling of structured support and direction, in the participants’ perceptions, was strengthened by the tools and strategies made available by the program throughout the process The accounts also suggest that the support experienced in the program was not limited to spontaneous social interactions but rather perceived as organized and consistent support within the proposed structure.

Recent studies indicate that social support—defined in the literature as the perception of emotional support and being welcomed, including elements such as listening, understanding, and encouragement [26,27], as well as instrumental or informational support, which involves practical guidance, assistance with problem solving, and information that promotes clarity [27]—is associated with lower levels of depressive symptoms. Although these studies suggest that perceived social support is associated with lower levels of depressive symptoms across different populations, the present study advances this discussion by examining this phenomenon within the context of a structured support system.

Unlike investigations that describe support as a spontaneous resource in the environment, in the present study, it was experienced by participants through tools developed exclusively for the program, such as the Protocols, Pense Comigo, PDA, and Plano Perfeito, as well as through weekly meetings and daily audio messages. In this context, perceived support was not limited to social interactions but also associated with a systematized set of resources and practices that, in the participants’ perceptions, contributed to reported changes in their depressive state.

The reconstruction of the way participants reported organizing their relationships throughout the program was associated, in their perceptions, with greater clarity regarding boundaries and self-responsibility in daily interactions. This can be understood within Permission Theory as a form of “relational intelligence”. In the literature, the more widely used concept is emotional intelligence, defined as the ability to perceive, understand, and regulate emotions in social interactions [28].

In the present study, the accounts indicated that participants came to perceive more clearly how they positioned themselves in everyday interactions, especially with regard to setting boundaries and managing their own decisions. Although the literature uses different concepts to describe modes of action in social relationships [29], the program’s proposal directs participants to observe concrete situations, identify behavioral and relational patterns, and adjust their way of acting based on their own criteria. In this way, the focus does not fall on isolated emotional aspects but rather the practical organization of interactions, which aligns with the changes reported by participants in how they conduct their relationships.

According to previous work by the authors [22], certain modes of action in relationships may occur without the person immediately recognizing them and may lead to interactions perceived as limiting, thereby favoring experiences of emotional dependence. In the theoretical structure that grounds the program, emotional dependence is defined as a mode of functioning in which a person’s state or action depends on the state or action of another and may occur in an active form—when one person influences or directs the emotional state of another—or in a passive form when the person allows themselves to be influenced or directed by the other. This definition helps to contextualize the accounts prior to participation in the program, in which participants mentioned fear, difficulty establishing boundaries, and challenges in positioning themselves in interactions, restricting their capacity to act according to their own criteria.

Participants reported that over the course of participation in the program, they came to perceive less patterns they associated with emotional dependence and greater permission to act and make decisions based on their own criteria. They mentioned greater security in defining boundaries, communicating needs, and assuming positions in everyday life, in contrast to previous experiences marked by fear or difficulty saying no. The expression “emotional prison”, used by one participant, illustrates the perception that emotional dependence limited permission to be who one is, to do what one wishes, and to exercise the right to succeed or fail—elements identified as important in this process of transformation. Overall, the accounts indicate that the reduction of emotional dependence, associated with perceived support and the development of awareness of one’s own interactions, was considered a relevant component in the reorganization of relationships and in the improvement of the depressive state [22,26,27].

The expression “usefulness of the illness” also describes how some participants came to interpret the presence of depression in their lives prior to entry into the program. In the theoretical structure that grounds the program, the process of becoming ill is conceptualized as an indicator that certain aspects of an individual’s life may require reorganization when the person does not consciously identify ways to deal with certain situations. This construct corresponds to the interpersonal secondary gain described in the literature [18,19], according to which the symptom may maintain relational configurations perceived as relevant.

Participants reported that upon understanding this perspective, they began to identify elements that, in their perceptions, were related to maintaining the difficulties they were experiencing and, based on this, felt more capable of reorganizing their decisions and actions. This process was described as fostering greater clarity regarding boundaries, needs, and choices, which, according to the participants themselves, was related to the perceived change in their depressive state, indicating the construction of a way of conducting their own lives that they considered more conscious, balanced, and oriented toward self-care and achieving goals.

Perceptions related to purpose and the future appeared in some accounts, as participants described greater clarity regarding personal and professional goals, resuming projects, and a feeling of direction during the program. These accounts indicate that processes of organization and monitoring goals were perceived as instrumental in structuring steps and resuming initiatives that had previously been interrupted. In addition, the support perceived in group interactions was cited as an element that contributed to maintaining the continuity of this movement. Group support and encouragement, as well as the Perfect Plan, daily audio messages, and weekly meetings, were also cited as elements that sustained this process.

Evidence from the literature indicates that planning strategies and collaborative goal setting foster greater initiative and active participation in decision-making and may contribute to a reduction in depressive symptoms [30]. In the present study, the reorganization of the future mentioned by participants aligns with the literature that links planning and goals to subjective perceptions of well-being.

Participants also highlighted that they changed the way they related to themselves, expressing greater responsibility for their actions, accompanied by attitudes oriented toward self-care, discipline, and a more favorable perception of their abilities and personal image. They reported greater willingness to adhere to routines, a reduction in harmful behaviors, and a re-engagement with regular physical activity. The literature suggests that regular physical activity is associated with a reduction in depressive symptoms, increased perceived self-efficacy, and improved daily functioning [9,31]. In addition, self-care may play an important role in the management of depressive symptoms and the promotion of overall well-being [1]. In the present study, these transformations were perceived by participants as part of a broader process of reorganization of everyday life, reflected in the way they structured their routines, intensified self-care practices, and began to manage time with greater clarity and consistency.

In addition to these transformations, participants also reported perceived changes in financial organization and professional performance during the program. These improvements included accounts of debt repayment, reorganization of economic life, and expansion of professional opportunities, consistent with evidence showing that occupational functioning is often impaired in depressive conditions [32,33] and tends to improve as symptoms decrease [33]. In the present study, these changes were reported by participants as occurring in parallel with other adjustments they were making to their routines and responsibilities such that the domains of health, career, and relationships were perceived as more organized and with greater clarity of direction.

The combination of greater routine organization, strengthening of interpersonal interactions, reduction of emotional dependence, increased permission to conduct aspects of one’s own life, greater clarity regarding goals and the future, and self-care practices was mentioned by participants as part of the set of adjustments made during the program, fostering relief, lightness, and a resumption of vitality. These elements, articulated with the perception of continuous support and structured direction, were reported as comprising the context in which participants perceived a reduction in the suffering associated with depression, indicating the relevance of considering different areas of life in the analysis of perceptions of change.

This study presents some limitations. The qualitative analysis was based on spontaneous accounts submitted by program participants, which implies the possibility of self-selection bias, since more engaged or more satisfied individuals may have a greater propensity to report their perceptions. Furthermore, as this involves retrospective and self-reported data, it was not possible to verify whether participants had a formal clinical diagnosis of depression or any formal record of such diagnosis or perceived severity, since the information analyzed did not include standardized assessments and reflected subjective perceptions of the depressive experience. The inclusion of the PHQ-9 as a standardized baseline screening instrument in future studies would allow a more rigorous assessment of the severity of self-reported symptoms and would facilitate comparisons with the literature.

Additionally, future implementations of the Program, or of similar initiatives addressing emotional distress, must mandatorily adopt a standardized baseline screening instrument, such as the PHQ-9. Participants with scores indicative of moderate to severe depression must be referred to licensed mental health professionals before or in parallel with participation, with formal documentation of this referral. Personal development programs do not replace specialized psychiatric or psychological treatment when clinically indicated, and the absence of screening and referral protocols represents an ethical risk that must be structurally corrected in future versions.

In addition to the self-selection bias inherent to voluntary participation, the analyzed reports derive from participants who consented to the public use of their testimonies and to the conducting of recorded interviews. Consequently, the sample may not fully represent the diversity of experiences present in the total set of Program participants.

Another limitation concerns the absence of standardization regarding the timing of report collection. Since participants could submit their testimonies spontaneously at any stage of participation, and the length of time in the Program was not recorded, it was not possible to assess how different stages of engagement may have influenced the analyzed reports.

The sample exclusively reflects participants who mentioned the topic, limiting the generalizability of the findings. Finally, the quantitative information reflects subjective perceptions about one’s own life and does not constitute clinical diagnostic measures.

Another relevant limitation of the present study is the absence of data on the sustainability of perceptions after program participation ends. Future studies should include follow-up assessments, preferably at six and twelve months post-program, to verify whether the perceived changes are maintained over time. This recommendation is supported by the literature: a meta-analysis on CBT demonstrated that therapeutic gains tend to be sustained at 6 to 12 months when participants continue to use self-monitoring strategies [11]. Studies on psychoeducational interventions have also documented sustainability of effects at four-week follow-up [12].

It is also important to consider the risk of social desirability bias in the analyzed accounts. The supportive environment of the program based on Permission Theory may have led participants to predominantly report positive transformations, omitting negative or ambivalent experiences. The literature documents that this bias is systematically underestimated: in formal clinical contexts, only approximately 9% of patients spontaneously disclose secondary motivations to the therapist [19]. In a group personal development program, this bias may be even more pronounced, given the component of group belonging and personal investment in the process. Future studies should incorporate methodological triangulation strategies, such as in-depth individual interviews with participants who discontinued the program and assessments by external informants, to mitigate this risk.

Despite these limitations, the findings contribute to understanding how participants describe their experiences before and during participation in the program, particularly with regard to perceptions related to depression.

Overall, participants reported perceived changes in different aspects of their lives during participation in the program, including references to a depressive experience perceived as less intense, a greater sense of well-being, and changes in relational aspects. These elements appeared in a distributed manner across the themes described by participants, composing the overall set of reported experiences. Such perceptions should be interpreted in light of the self-reported nature of the accounts, without causal inference, but they offer an overview of the ways participants understood and narrated their experiences throughout the analyzed period.

Although the present study does not allow for the identification of which specific components of the Program contributed to the perceived changes, it is plausible to assume that such experiences result from the interaction among multiple elements of the proposal. Among these, the following stand out: the self-reflection process associated with the construction of the Perfect Plan, the protocols grounded in Permission Theory (Emotional Dependence, Emotional Protection, Fear, and Guilt) aimed at identifying and reorganizing relational patterns, the PDA cycle (Perceive, Decide, and Act), which emphasizes personal responsibility and the implementation of concrete actions, and the social support offered by the community of participants. From this perspective, the reported benefits may reflect the combination of these different processes acting in an integrated manner, rather than the isolated action of a single component. However, this interpretation remains hypothetical, since the study design was not conceived to assess causal mechanisms or to estimate the relative contribution of the different elements of the Program.

## 9. Conclusions

In this study, participants reported that their experience of the program based on Permission Theory was associated not only with perceived changes in their depressive experience but also with transformations in different aspects of life, including improvement in interpersonal relationships, a reduction in emotional dependence, and an increase in permission to be, have, and act based on their own criteria. These perceptions aligned with reported improvements across the three pillars proposed by the program—career/finances, relationships with oneself and with others, and physical and mental health—including a perceived reduction in depressive symptoms, a central aspect of this study’s objective.

Although the results point to consistent perceptions of change, future studies—especially randomized controlled trials—are necessary to examine, in a controlled manner, the effects of the program on depressive symptoms and other health indicators. Such investigations may deepen our understanding of the processes involved in the changes perceived by participants and assist in consolidating the scientific basis of this approach.

## Figures and Tables

**Table 1:** Matrix of the relational utility of the impactful event.

Consequence vs. Effect	Uniting	Distancing
Sparing	Event avoids a cost/responsibility and brings closer	Event avoids a cost/responsibility and generates distancing
Forcing	Event is painful, but brings closer	Event is painful and generates distancing

Source: elaborated by Elton Euler.

**Table 2:** Summarized description of the protocols used in the Program.

Protocol	Main Objective	Main Components
Emotional Dependence	To facilitate the identification of patterns of excessive interference in interpersonal relationships and to promote the establishment of relational boundaries.	Identification of relational patterns, differentiation between help and support, analysis of intentions and expectations, assessment of harm resulting from interference, definition of boundaries, relational agreements, repair of harm, and planning of concrete actions.
Emotional Protection	To assist in preventing relapse into patterns of emotional dependence and in managing persistent relational interference.	Identification of recurring controlling patterns, analysis of relational expectations and agreements, assessment of the costs of maintaining interference, recognition of recurring relational errors, and definition of new strategies for interaction and boundary protection.
Fear	To promote reflection on fears that influence decisions and behaviors, facilitating the analysis of alternatives and conscious decision-making.	Identification of the origin and impact of fear, analysis of associated beliefs, assessment of the costs and consequences of avoidance, comparison of alternative perspectives, mapping of possible futures, and elaboration of an action plan based on the PDA cycle (Perceive, Decide, and Act).
Guilt	To facilitate the analysis of feelings of guilt associated with interpersonal events and the attribution of responsibility.	Identification of the predominant guilt, assessment of the related event, evaluation of perceived impacts, distinction between facts and interpretations, analysis of one’s own and others’ responsibilities, reflection on attributions of responsibility, and identification of possible undue transfers or extensions of responsibility.

Source: elaborated by the authors.

**Table 3:** Content analysis of participants’ self-reports.

Context Unit	Registration Unit	Categories
Before program participation	Professional and financial issues	Financial instability and precarious work
	Relational issues	Conflictual interpersonal relationships and lack of boundaries High emotional dependence and lack of permission
	Health and self-esteem	Perceptions related to depression Impaired physical health and risk behaviors Low self-esteem and negative self-image
	Health and self-esteem	Absence of purpose and life perspectives
During program participation	Professional and financial issues	Financial and professional improvement
	Relational issues	Reconstruction of relational stance and self-care Increased permission and decreased emotional dependence Feeling of support and direction Understanding the usefulness of illness
	Health and self-esteem	Perceptions of reduction of the depressive experience
	Life perspective	Planning and future projection

Source: elaborated by the authors.

**Table 4:** Sociodemographic characteristics of the participants (*n* = 23).

Variable	*n*	%
Sex		
Female	18	78.3
Male	5	21.7
Age group		
≤40 years	8	34.8
41 to 50 years	8	34.8
>50 years	7	30.4
Marital status		
Married	12	52.2
Divorced	7	30.4
Single	4	17.4
Current employment status		
Homemaker	2	8.7
Entrepreneur/business owner	8	34.8
Self-employed professional	4	17.4
Independent worker	8	34.8
Formal employee (CLT)	1	4.3
Current group		
Central–west	1	4.3
Northeast	3	13.1
North	1	4.3
Southeast	11	47.8
South	6	26.2
USA	1	4.3
Pillar with best result		
Financial	8	34.8
Relationships	7	30.4
Health	8	34.8
Total	23	100.0

CLT: Consolidation of Labor Laws (Brazilian labor code). Source: elaborated by the authors.

**Table 5:** Life classification before and during the program.

Descriptive Measures	Score Before the Program	Score After the Program	Difference After–Before	*p*-Value *
Mean (SD)	3.1 (1.7)	8.7 (1.0)	5.5 (2.2)	0.000
95% CI	2.4–3.9	8.2–9.1	4.8–6.5	
Median	3.0	9.0	5.0	
Min–Max	1.0–7.0	07–10	2.0–9.0	

SD: standard deviation; 95% CI: 95% confidence interval; Min: minimum value; Max: maximum value; * Wilcoxon test. Source: elaborated by the authors.

**Table 6:** Comparison of differences (before–during) in life classification according to sociodemographic characteristics.

Variable	Difference (During–Before) Average (DP)	*p*-Value
Sex		0.587 *
Female	5.4 (2.1)	
Male	6.0 (2.7)	
Age group		0.893 **
≤40 years	5.6 (2.2)	
41 to 50 years	5.9 (2.7)	
>50 years	5.0 (2.2)	
Marital status		0.563 **
Married	5.8 (1.8)	
Divorced	5.4 (2.8)	
Single	4.8 (2.5)	
Pillar with best result		0.375 **
Financial	6.1 (2.4)	
Relationships	5.7 (2.0)	
Health	4.8 (2.2)	
Total	23	100.0

SD: standard deviation; * Mann–Whitney test, ** Kruskal–Wallis test. Source: elaborated by the authors.

## Data Availability

The raw data generated and analyzed in this study cannot be made publicly available due to ethical and confidentiality restrictions, as they contain sensitive personal reports provided by the research participants. However, the data are available to the journal reviewers during the peer review process and may be accessed upon request from the corresponding author, subject to appropriate ethical and confidentiality safeguards.
